# Application of self-made new endoscopic sleeve guided by wire drainage tube in minimally invasive operation of supratentorial deep brain hematoma

**DOI:** 10.1038/s41598-025-22418-z

**Published:** 2025-11-04

**Authors:** Jingling Qiang, Congkun Tian, Mengmeng Ren, Hanlei Duan, Yinsong Yuan, Xiaogang Yang, Yongjun Dong

**Affiliations:** 1 Department of Neurosurgery, Affiliated Hospital of Yan’an University, Yan’an, 716000 Shaanxi China; 2 Department of Neurosurgery, 3201 Hospital, Hanzhong, China; 3 Department of Neurosurgery, Yan’an Hospital of Traditional Chinese Medicine, Yan’an, China

**Keywords:** Self-made guide-wire drainage tube, Novel endoscopic sleeve, Supratentorial deep-seated hemorrhage, Minimally invasive surgery, Clinical efficacy, Diseases, Medical research, Neurology, Neuroscience

## Abstract

This study aimed to examine the clinical effectiveness of a new endoscopic sleeve guided by a custom-made drain and guidewire in minimally invasive surgery for supratentorial deep intracerebral hematoma. This study included 168 individuals diagnosed with supratentorial deep cerebral hemorrhage between January 2019 and January 2023. Patients were divided into two groups based on their treatment plans: those who had hematomas removed using a new endoscopic sleeve guided by a homemade drainage tube with guidewire were included in the Experimental group(*n* = 84), and those who had hematomas removed using a cranial microscope with a small bone window were included in the control group. Preoperative indices, surgical indices, postoperative complications, discharge outcomes, and prognosis were compared between the two groups. The results found that there were no statistically significant differences between the patients in terms of sex, age, hemorrhage site, preoperative hemorrhage volume (ml), preoperative GCS score, hospital stay, intracranial infection, and epilepsy (*P* > 0.05). The experimental group was compared with the control group in terms of operative time, intraoperative blood loss, postoperative residual hematoma volume, hematoma clearance rate, and postoperative hematoma-related outcomes. There was a significant difference (*P* < 0.05) in GCS score at discharge, Rankin score at discharge, and GOS score at 3 months after surgery.The new homemade endoscopic sleeve can reduce collateral damage to brain tissue during sleeve placement, reduce postoperative bleeding from the endoscopic working channel and rebleeding in the operative area, increase the hematoma clearance rate, shorten the operative time, and make endoscopic hematoma removal surgery more precise and minimally invasive.

## Introduction

Hemorrhagic stroke is a disease with high mortality and disability rates. With societal aging and increased use of antiplatelet drugs, its proportion among all stroke patients has gradually risen (9%−28%), making it the second leading cause of death globally^1^. Basal ganglia and thalamic hemorrhages are common types of supratentorial deep-seated hemorrhagic stroke, associated with high mortality and poor prognosis^2^. Over the years, despite extensive research and considerable progress in the surgical treatment of supratentorial deep-seated hemorrhage, the optimal strategy and method remain controversial^3^. Traditional small bone window craniotomy under microscopy, while allowing direct visualization and hematoma evacuation, is associated with longer operative times, greater intraoperative blood loss, more extensive brain exposure, and higher risk of postoperative complications such as rebleeding and infection^4,5^. Advances in neuroendoscopic equipment, improved endoscopic techniques, and the pursuit of minimally invasive surgery have established neuroendoscopic evacuation as a promising alternative for supratentorial deep-seated hemorrhage^6,7^. This technique involves minimally invasive surgery using a sleeve. After imaging-based hematoma localization, brain tissue is dilated, and a sleeve of appropriate length and size is inserted into the hematoma cavity. The neuroendoscope is then introduced through the sleeve to visualize the hematoma and bleeding points clearly, allowing instruments to evacuate the hematoma and achieve hemostasis^8^. However, the key steps in this procedure are preoperative localization and sleeve insertion. This paper introduces the application of a novel self-made endoscopic sleeve guided by a guide-wire drainage tube in minimally invasive surgery for supratentorial deep-seated hematoma and compares its efficacy with traditional small bone window craniotomy under microscopy.

## Materials and methods

### Patient population

A total of 168 patients with supratentorial deep-seated intracerebral hemorrhage, admitted to the Department of Neurosurgery at the Affiliated Hospital of Yan’an University between January 2019 and January 2023, were included. Patients were divided into two groups: the experimental group (*n* = 84) underwent hematoma evacuation using the novel self-made endoscopic sleeve guided by a guide-wire drainage tube, while the control group (*n* = 84) underwent hematoma evacuation via small bone window craniotomy under microscopy. All patients or their families provided written informed consent. Observed indicators included:

1. Preoperative: Gender, age, hemorrhage location, preoperative hematoma volume (ml), preoperative GCS score.

(2) Surgical: Operative time (min), intraoperative blood loss (ml), postoperative residual hematoma volume (ml), hematoma clearance rate (%), postoperative drainage time (h).

(3) Postoperative Complications: Surgical site rebleeding, tracheotomy, pulmonary infection, intracranial infection, epilepsy.

(4) Outcomes & Prognosis: Discharge GCS score, discharge modified Rankin Scale (mRS) score, length of hospital stay (d), GOS score at 3 months post-discharge.

Operative time was defined from skin incision to anesthesia end (minutes). Postoperative residual hematoma volume and clearance rate were calculated using 3D-Slicer software^9^ for segmentation on CT scans performed within 24 h postoperatively. Postoperative drainage tube duration was recorded in hours. Blood pressure was strictly controlled postoperatively. Follow-up assessments (GOS) were conducted 3 months after discharge.

#### Inclusion criteria

(1) Hypertension diagnosis confirmed; basal ganglia or thalamic hemorrhage verified by head CT and head/neck CTA; (2) Age > 18 years; (3) Hematoma volume > 30 ml, located supratentorially (basal ganglia or thalamus); (4) GCS score > 4, no brain herniation, stable vital signs.

#### Exclusion criteria

(1) Hemorrhage secondary to aneurysm, intracranial arteriovenous malformation, tumor, hemorrhagic transformation of infarction, long-term anticoagulant use, or other diseases; (2) Severe pre-existing cardiac, pulmonary, hepatic, or renal insufficiency; (3) Loss to follow-up.

### Principle and technique

#### Principle

 Based on preoperative CT reconstruction for anatomical localization, the guide-wire drainage tube was inserted to aspirate dark blood, confirming its tip was within the hematoma cavity. Utilizing brain tissue compliance, sequential dilation was performed: first with the primary dilator sleeve, then with the secondary dilator working sleeve. Specifically, the primary dilator sleeve was advanced over the drainage tube into the hematoma cavity. The secondary working dilator sleeve was then advanced over the primary sleeve into the cavity. Upon confirming correct placement of the working sleeve within the hematoma cavity, the guide-wire drainage tube and primary dilator sleeve were removed. The neuroendoscope was introduced through the working sleeve for direct visualization during hematoma evacuation and hemostasis.

##### Materials

A standard F12 cranial external drainage tube with guide-wire (outer diameter 4.0 mm) was used. Independently designed and manufactured dilators (Table 1).

**Table 1:** Specifications of self-made dilator sleeves.

Type	Tip Opening (mm)	Inner Diameter (mm)	Outer Diameter (mm)	Wall Thickness (mm)	Total Length (cm)	Material
Primary Dilator Sleeve	4.5	5.0	7.0	1.0	12.0	Glass
Secondary/ Working Dilator Sleeve	8.0	9.0	11.0	1.0	10.0	Glass

All sleeves featured graduated markings.

The self-made endoscopic sleeve material (glass) is reusable after high-pressure autoclave sterilization preoperatively, incurring no additional medical costs.

##### Surgical technique (experimental group)

All patients underwent general anesthesia. Preoperative CT and skin mark localization determined the puncture point, trajectory, depth, and target, avoiding major fiber tracts and targeting the hematoma long axis to minimize white matter tract transection^10^.The insertion trajectory (parallel to the midline toward the lateral canthus at a depth of 8–9 cm for posterolateral thalamic hematomas) was consistent in principle but adjusted based on individual hematoma location and size as determined by preoperative CT.

###### Basal ganglia or anteromedial thalamic hematoma

**Fig. 1:**
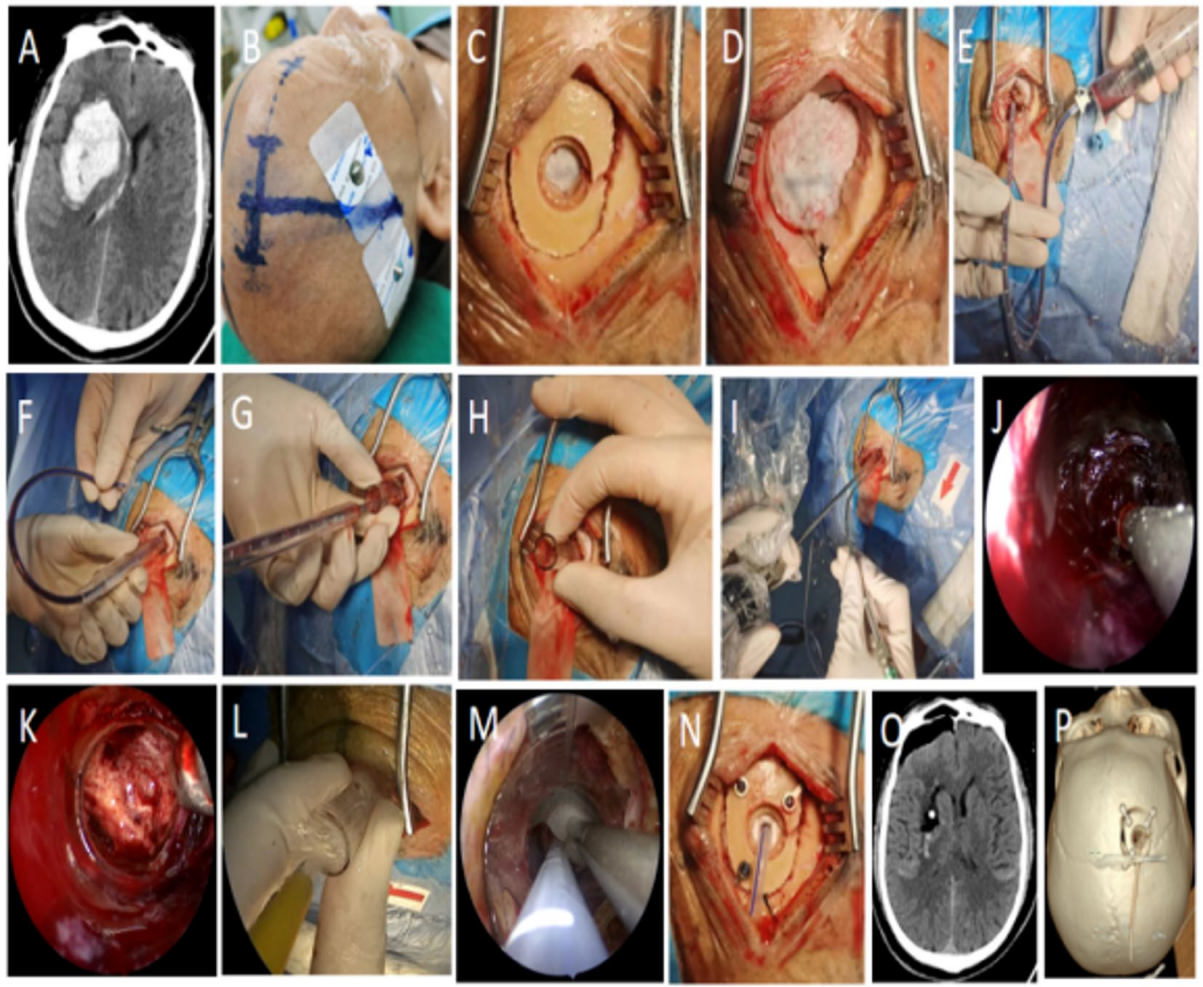
Surgical procedure for right basal ganglia hemorrhage. (**A**) Head CT showing right basal ganglia hemorrhage. (**B**) Surgical path planning based on head CT and skin mark. (Skin marker location was decided based on preoperative CT reconstruction, marking the entry point and trajectory on the scalp to guide the insertion angle and depth.) (**C-D**) Surgical incision 5 cm and bone window 2.5 cm x 3.0 cm. (**E**) Guide-wire drainage tube aspiration of dark blood confirming position within hematoma cavity; 10–20 ml aspirated for gradual ICP reduction. (**F-G**) Sequential insertion of primary and secondary dilator sleeves over the drainage tube; subsequent removal of drainage tube and primary sleeve. (**H-I**) Secondary/Working sleeve in place; neuroendoscope and suction introduced. (**J-K**) Hematoma evacuation; exposed white matter after evacuation. (**L-M**) Irrigation via working sleeve; no active bleeding observed; hemostatic material placed under endoscopic vision; drainage tube placement. (**N**) Bone flap replacement and fixation. (**O-P**) Postoperative head CT showing satisfactory evacuation of right basal ganglia hematoma; 3D reconstruction showing right frontal keyhole approach.

 Employed a frontal small bone window keyhole approach. Surgical planning: puncture point located 2 cm anterior to the coronal suture and 3 cm lateral to the midline. A 5–6 cm linear scalp incision parallel to the midline was made. After retraction, a 3.0 cm x 2.5 cm bone window was created centered 2 cm anterior to the coronal suture. Dura was opened in a cruciate fashion. The guide-wire drainage tube was inserted parallel to the sagittal plane towards the hematoma target (6–7 cm deep from cortex). The guide-wire was removed, and 10–20 ml of dark blood was slowly aspirated to achieve gradual intracranial pressure (ICP) reduction. The guide-wire was reinserted. Utilizing brain compliance, the primary then secondary working dilator sleeves were sequentially advanced over the drainage tube. Saline irrigation was used during dilation to reduce friction. Upon confirming the working sleeve within the hematoma cavity, the drainage tube and primary sleeve were removed. A 4 mm diameter, 0° rigid endoscope (Karl Storz, Germany) was used. A matching endoscopic suction device (with a straight tip and curved distal end) was employed for hematoma evacuation in hard-to-reach areas, and hemostasis was achieved using bipolar electrocautery. Hematoma evacuation and hemostasis were performed under endoscopic visualization. After satisfactory evacuation and hemostasis, a drainage tube was placed under endoscopic vision. Gelatin sponge was packed around the tube entry site. The working sleeve was withdrawn under vision. Dura was sutured, bone flap replaced, and the surgical incision was closed (Fig. 1).

###### Posterolateral thalamic hematoma

**Fig. 2:**
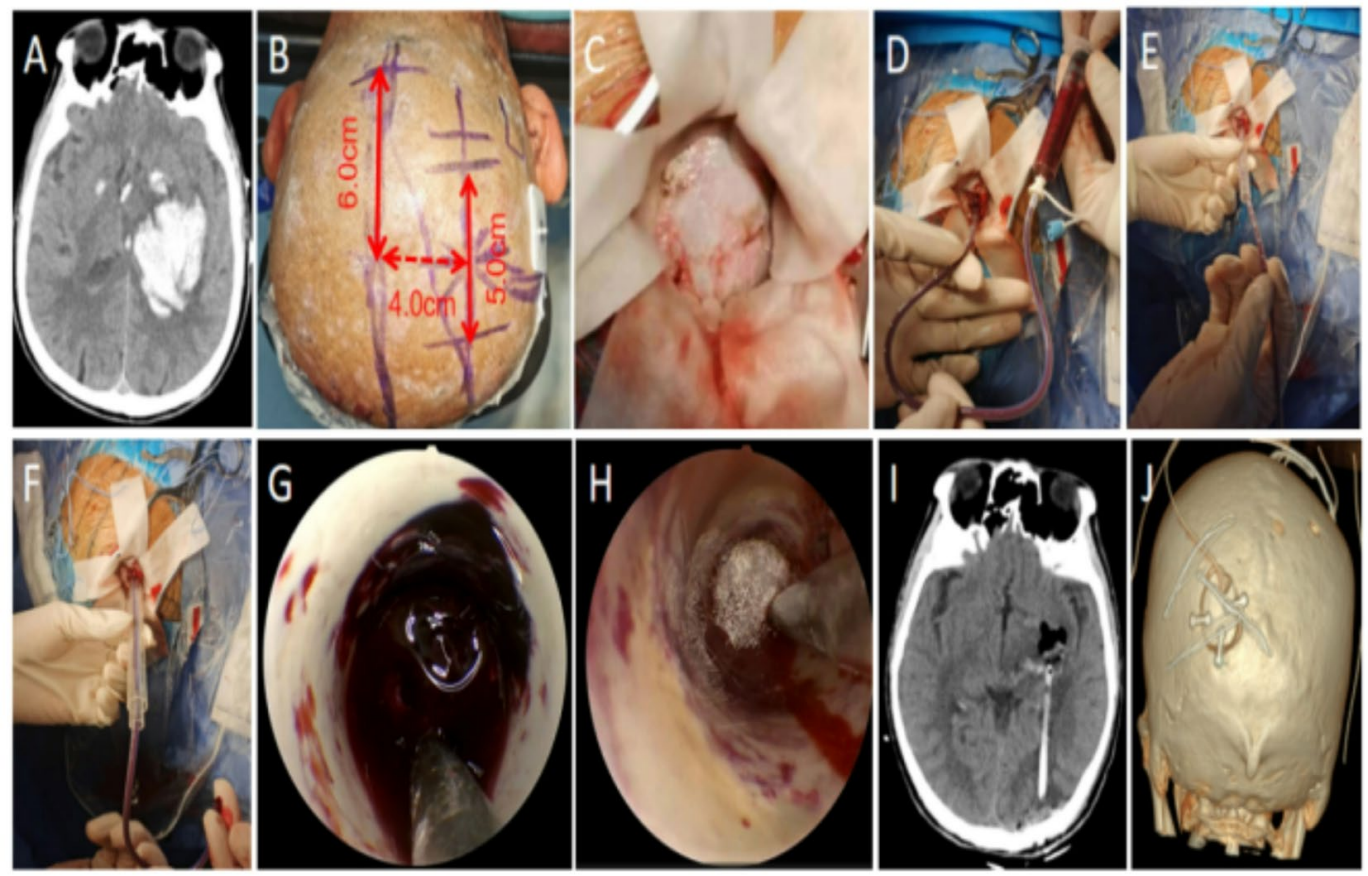
Surgical procedure for left posterolateral thalamic hemorrhage. (**A**) Head CT showing left posterolateral thalamic hemorrhage. (**B**) Surgical path planning based on head CT and skin mark. (Skin marker location was decided based on preoperative CT reconstruction.) (**C**) Surgical incision 5 cm and bone window 2.5 cm x 3.0 cm. (**D-F**) Guide-wire drainage tube aspiration confirming position within hematoma cavity; 10–20 ml aspirated; sequential insertion of primary and secondary dilator sleeves; removal of drainage tube and primary sleeve. (**G-H**) Endoscopic hematoma evacuation; hemostatic material covering cavity post-evacuation. (**I-J**) Postoperative head CT showing satisfactory evacuation of left thalamic hematoma; 3D reconstruction showing left occipital keyhole approach.

Employed an occipital small bone window keyhole approach (prone position, Mayfield head fixation). Puncture point: 4 cm lateral to midline and 6 cm superior to the external occipital protuberance. A 5–6 cm linear scalp/muscle incision parallel to the sagittal plane was made. A 3.0 cm x 2.5 cm bone window was created. The guide-wire drainage tube was inserted parallel to the midline towards the lateral canthus 8–9 cm deep. Subsequent dilation and endoscopic evacuation proceeded as described above. Active bleeding points were coagulated using bipolar electrocautery(Fig.2). For ICH cases with intraventricular hematoma extension (*n* = 12 in experimental group, *n* = 15 in control group), a contralateral external ventricular drain was placed routinely.

###### Surgical technique (control group)

Based on preoperative anatomical imaging, a linear or curvilinear incision was made. A 4 cm x 5 cm small bone window was created. Hematoma evacuation was performed under microscopy via transsylvian-transinsular approach or the shortest transcortical approach to the hematoma. Dura was sutured, bone flap replaced and fixed, a drainage tube was routinely placed in the hematoma cavity, and the surgical incision was closed^11^. Two patients in the control group required large bone flap removal due to severe postoperative cerebral edema and refractory intracranial hypertension; decompressive craniectomy was performed. In the experimental group, sufficient brain relaxation and ICP normalization were achieved in all cases, and no patient required decompressive craniectomy. Patients with intraventricular extension also received a contralateral ventricular drain.

### Statistical analysis

Statistical analysis was performed using SPSS 23.0 software. Normally distributed continuous data were compared using independent samples t-tests and expressed as mean ± standard deviation (SD). Non-normally distributed continuous data were compared using non-parametric tests (Mann-Whitney U) and expressed as median (interquartile range, IQR). Categorical data were compared using Chi-square tests or Fisher’s exact test. A *P* < 0.05 was considered statistically significant.

## Results

### Preoperative characteristics

No significant differences were found between the experimental and control groups in gender, age, hemorrhage location, preoperative hematoma volume, preoperative GCS score, or length of hospital stay (*P* > 0.05, Table 2).

**Table 2:** Comparison of preoperative baseline characteristics between groups.

Characteristic	Experimental Group (*N* = 84)	Control Group (*N* = 84)	t/χ²	*P*
Gender (Male)	54 (64.3%)	51 (60.7%)	0.229	0.750
Age (years)	57.92 ± 12.36	56.13 ± 11.24	0.979	0.329
Hemorrhage Location			1.294	0.729
Left Basal Ganglia	33 (39.3%)	29 (34.5%)		
Right Thalamus	7 (8.3%)	9 (10.7%)		
Right Basal Ganglia	35 (41.7%)	33 (39.3%)		
Left Thalamus	9 (10.7%)	13 (15.5%)		
Preop Hematoma Vol (ml)	47.07 ± 8.79	48.03 ± 9.48	−0.680	0.497
Preop GCS Score	8.26 ± 1.83	8.30 ± 2.06	−0.119	0.906
Hospital Stay (day)	16.01 ± 4.17	16.18 ± 4.39	−0.252	0.801

### Surgical outcomes

Significant differences favoring the experimental group were observed in operative time, intraoperative blood loss, postoperative residual hematoma volume, hematoma clearance rate, and postoperative drainage time (*P* < 0.05, Table 3).

**Table 3:** Comparison of surgical outcomes between groups.

Outcome Measure	Experimental Group (*N* = 84)	Control Group (*N* = 84)	Z	*P*
Operative Time (min)	95 (90, 110)	155 (140, 175)	−11.012	< 0.001
Intraop Blood Loss (ml)	60 (50, 80)	170 (150, 190)	−11.220	< 0.001
Postop Residual Vol (ml)	4.00 (2.89, 5.23)	7.79 (4.62, 10.29)	−6.440	< 0.001
Clearance Rate (%)	91.31 (88.50, 93.63)	83.50 (79.74, 88.68)	−7.077	< 0.001
Drainage Time (h)	39.00 (30.00, 44.75)	41.00 (28.00, 50.75)	−2.033	0.042

### Postoperative complications

The experimental group had significantly lower rates of surgical site rebleeding, tracheotomy, and pulmonary infection compared to the control group (*P* < 0.05). No significant differences were found in intracranial infection or epilepsy rates (*P* > 0.05, Table 4).

**Table 4:** Comparison of postoperative complications between groups.

Complication	Experimental Group (*N* = 84)	Control Group (*N* = 84)	χ²	*P*
Surgical Rebleeding	2 (2.4%)	10 (11.9%)	5.774	0.032
Tracheotomy	3 (3.6%)	11 (13.1%)	4.987	0.047
Pulmonary Infection	4 (4.8%)	13 (15.5%)	5.301	0.038
Intracranial Infection	2 (2.4%)	4 (4.8%)	-	0.628
Epilepsy	4 (4.8%)	5 (6.0%)	-	> 0.999

### Discharge outcomes and prognosis

Significant differences favoring the experimental group were observed in discharge GCS score, discharge mRS score, and GOS score at 3 months postoperatively (*P* < 0.05, Table 5).

**Table 5:** Comparison of discharge outcomes and prognosis between groups.

Outcome Measure	Experimental Group (*N* = 84)	Control Group (*N* = 84)	Z	*P*
Discharge GCS Score	11 (10, 13)	10 (9, 12)	−2.808	0.005
Discharge mRS Score	3 (2, 3)	3 (2, 4)	−2.380	0.017
3-month GOS Score	4 (3, 4)	3 (2, 4)	−5.896	< 0.001

## Discussion

Intracerebral hemorrhage (ICH) is the most common form of hemorrhagic stroke, characterized by high morbidity and mortality. Rapid and massive bleeding compresses brain tissue, causing mass effect and pathological sequelae such as reduced local cerebral blood flow, acidosis, inflammation, and immune responses^12^. Surgical evacuation directly removes the hematoma, reduces intracranial pressure (ICP), and alleviates pressure on the brain and neurovascular structures. Beyond mitigating mass effect, blood removal also reduces toxic stimuli from hematoma breakdown and inflammatory cascades in brain tissue, thereby attenuating secondary brain injury^13^.

Traditional small bone window craniotomy under microscopy has been a standard surgical approach for ICH evacuation, offering direct visualization and control. However, it is associated with longer operative times, greater tissue exposure, higher intraoperative blood loss, and increased risk of complications such as rebleeding and infection^14^. Minimally invasive surgical approaches are increasingly utilized in ICH management. Neuroendoscopic hematoma evacuation is now recognized as a safe and effective procedure, offering advantages of minimal trauma, fewer complications, and faster postoperative recovery^15^. Latest research continues to support the efficacy of endoscopic evacuation compared to craniotomy, particularly in reducing operative time and improving functional outcomes^16,17^.

The guide-wire-assisted sleeve insertion method described herein offers significant advantages compared to traditional small bone window craniotomy under microscopy: First, it minimizes brain exposure and tissue disruption. Craniotomy requires a larger bone window and brain retraction to access the hematoma, potentially damaging eloquent areas. Our technique uses a precise, small-diameter working channel created with graded dilation, minimizing collateral damage. Second, the endoscopic approach provides superior illumination and magnification of the hematoma cavity, facilitating more complete evacuation and identification of bleeding points compared to the microscopic view through a limited corridor^18^. Third, directly inserting a large working sleeve into the brain under high ICP increases intracranial volume and pressure, worsening injury. Our technique first aspirates 10–20 ml of blood via the drainage tube, allowing gradual ICP reduction before graded sleeve insertion. The use of a 10–20 mL syringe for aspiration applies controlled negative pressure. While larger syringes can generate higher negative pressures, potentially risking parenchymal injury or rebleeding, the slow, controlled aspiration under continuous visual confirmation of dark blood return and the immediate cessation upon encountering resistance were key safety measures in our study. Reduced local pressure around the hematoma also promotes retraction of surrounding brain tissue, facilitating hematoma movement towards the channel, reducing the need for sleeve manipulation and further minimizing injury^19^. Fourth, the outer diameter of our sleeve (11 mm) is significantly smaller than the exposure required for microscopic surgery, further reducing tissue damage.

In this study, the self-made endoscopic sleeve technique achieved a significantly higher hematoma clearance rate (91.31% vs. control, *P* < 0.05), shorter operative time (95 min vs. control, *P* < 0.05), and lower rebleeding rate (2.4% vs. control, *P* < 0.05) compared to small bone window craniotomy under microscopy. Significant improvements were also seen in discharge GCS, discharge mRS, and 3-month GOS scores (*P* < 0.05). Reduced complications significantly improved patient prognosis. The findings demonstrate the superiority of this novel endoscopic technique over the traditional microscopic craniotomy approach in the studied parameters. Additionally, this technique allowed evacuation of larger hematomas, expanding the indications for endoscopic surgery. We successfully applied it in selected patients with impending neurological deterioration due to mass effect, performing endoscopic evacuation combined with contralateral ICP monitoring. The inclusion criteria formally excluded patients with established brain herniation; however, in clinical practice, the technique was cautiously applied in a few select cases with impending herniation after careful consideration and institutional approval. If brain collapse was adequate (cortex > 1 cm from inner table) and ICP normalized (< 20 mmHg), decompressive craniectomy was avoided; otherwise, it was performed by extending the incision. In both cases of surgical rebleeding, postoperative cranial CT scans were compared with preoperative ones to distinguish between active bleeding and residual blood clots. The hematoma volume in both rebleeding cases was relatively small. Urokinase was injected through the hematoma cavity drainage catheter to dissolve the blood clots, followed by continuous drainage. The intracranial hematomas were gradually absorbed and dissipated.

The working sleeve was initially positioned at the center of the hematoma based on preoperative planning to facilitate access to all parts of the clot. In cases where the initial placement was deemed suboptimal (e.g., too superficial), the accompanying endoscopic suction device (with a straight tip and curved distal end) was used to carefully evacuate deeper blood clots under direct visualization. Brain prolapse after superficial evacuation was managed by adjusting the patient’s position (head elevation), ensuring adequate anesthesia depth, and using careful irrigation and suction to maintain visualization of deeper cavities.

Navigation technology was not employed in any cases in this series, relying solely on preoperative CT-based planning and anatomical landmarks.

This study has several limitations. Its retrospective design and single-center setting may introduce selection bias and limit generalizability. The follow-up period was relatively short (3 months), and longer-term outcomes are unknown. The technique requires a learning curve for proficiency in endoscopic manipulation within the confined working space (9 mm inner diameter). Practical tips for effective operation within this space include proficient use of instruments matched with the endoscope, meticulous hemostasis, frequent irrigation, and careful coordination between the endoscopist and assistant.Reproducibility in other centers needs further validation. Furthermore, while future applications in tumor or aneurysm surgery are hypothesized, similar tubular retractor systems have been previously described for minimally invasive evacuation and tumor surgery^20,21^, and these contributions should be acknowledged.

In conclusion, the application of this novel self-made endoscopic sleeve guided by a guide-wire drainage tube in minimally invasive surgery for supratentorial deep-seated hematoma showed significant advantages over traditional small bone window craniotomy under microscopy, including reduced iatrogenic brain and vascular injury during access, decreased postoperative rebleeding, increased hematoma clearance rate, shortened operative time, and improved patient prognosis, enhancing the precision and minimally invasive nature of hematoma evacuation. However, this technique demands higher proficiency in endoscopic manipulation due to the limited working space (9 mm inner diameter). Future developments may involve graded dilation to larger diameters using this system, potentially facilitating deep-seated tumor resection or even aneurysm clipping.

## Data Availability

The datasets generated during and/or analyzed during the current study are available from the corresponding author on reasonable request.

## Abbreviations

CT: computed tomography
GCS: glasgow coma scale
GOS: glasgow outcome scale
ICH: intracerebral hemorrhage
ICP: intracranial pressure
